# Mechanics, thermodynamics, and kinetics of ligand binding to biopolymers

**DOI:** 10.1371/journal.pone.0174830

**Published:** 2017-04-05

**Authors:** Javier Jarillo, José A. Morín, Elena Beltrán-Heredia, Juan P. G. Villaluenga, Borja Ibarra, Francisco J. Cao

**Affiliations:** 1 Departamento de Física Atómica, Molecular y Nuclear. Facultad de Ciencias Físicas. Universidad Complutense de Madrid. Pza. de las Ciencias, 1. Madrid. Spain; 2 Instituto Madrileño de Estudios Avanzados en Nanociencia (IMDEA Nanociencia) & CNB-CSIC-IMDEA Nanociencia Associated Unit ‘Unidad de Nanobiotecnología’, Madrid, Spain; 3 Departamento de Física Aplicada I. Facultad de Ciencias Físicas. Universidad Complutense de Madrid. Pza. de las Ciencias, 1. Madrid. Spain; Semmelweis Egyetem, HUNGARY

## Abstract

Ligands binding to polymers regulate polymer functions by changing their physical and chemical properties. This ligand regulation plays a key role in many biological processes. We propose here a model to explain the mechanical, thermodynamic, and kinetic properties of the process of binding of small ligands to long biopolymers. These properties can now be measured at the single molecule level using force spectroscopy techniques. Our model performs an effective decomposition of the ligand-polymer system on its covered and uncovered regions, showing that the elastic properties of the ligand-polymer depend explicitly on the ligand coverage of the polymer (i.e., the fraction of the polymer covered by the ligand). The equilibrium coverage that minimizes the free energy of the ligand-polymer system is computed as a function of the applied force. We show how ligands tune the mechanical properties of a polymer, in particular its length and stiffness, in a force dependent manner. In addition, it is shown how ligand binding can be regulated applying mechanical tension on the polymer. Moreover, the binding kinetics study shows that, in the case where the ligand binds and organizes the polymer in different modes, the binding process can present transient shortening or lengthening of the polymer, caused by changes in the relative coverage by the different ligand modes. Our model will be useful to understand ligand-binding regulation of biological processes, such as the metabolism of nucleic acid. In particular, this model allows estimating the coverage fraction and the ligand mode characteristics from the force extension curves of a ligand-polymer system.

## I. Introduction

The study of biopolymers interactions with small ligands is an essential topic to many areas of research. Biological systems abound with polymers such as polynucleotides or polysaccharides, and ligands such as proteins, metal ions, antibiotics, drugs, among others. Thus, there are numerous structural, biochemical and thermodynamic studies on the binding of proteins to nucleic acids [1–22]. The binding of multivalent ions, oligolysines or oligopeptides to polynucleotides has also been studied in depth [10,23–29].

Recently, single-molecule manipulation experiments have led to a significant progress in understanding the fundamental properties of a variety of polymers [30]. Essentially, single-molecule experiments measure the end-to-end extension of a single polymer molecule stretched under controlled force. The resulting extension versus force data are related to microscopic physical properties of the polymers by using some idealized models. Typical models are the freely-jointed chain (FJC) model, where the polymer consists of rigid Kuhn segments of a given length connected by flexible joints, and the worm-like chain (WLC) model, where the polymer is modeled as a continuous elastic thin rod [31,32]. The FJC and WLC models account for the polymer’s local bending stiffness, but they ignore the interactions between monomers well separated along the chain, the so-called excluded-volume interaction. These models successfully describe the elastic properties of synthetic and biological polymers. In particular, the elasticity of ds- and ss- DNA molecules at low ionic strength conditions (i.e. below 50 mM NaCl) at intermediate-force regimes, where the applied force effectively screens long-range monomer interactions. However, these models are not valid to explain the elastic properties of ssDNA at low forces and higher ionic conditions (i.e. above 100 mM NaCl), probably due to the formation of transient secondary structures in these nucleic acid molecules. Extensions of the FJC and WLC models have been formulated to account for the low-force elasticity of ssDNA (snake-like chain model, SLC model [33]), the base-pairing and base-pair stacking interactions [34–36]. Thick Chain (TC) model [37–38] and scaling model of non-ideal polymer under tension [33,39–41] have been successfully applied to model the elastic properties of flexible charged biopolymers.

Single-molecule force spectroscopy experiments have also been extended to study a number of proteins that bind dsDNA, ssDNA, or both [4,8,9,42–48], and the resulting elastic properties of these protein-DNA complexes

Henceforth, a first step in the study of the binding processes is to characterize the changes caused by the bound ligands on the elastic properties of the polymer, which in turn, can be used to obtain a deeper insight in the binding interactions between ligands and biopolymers. Here, we propose a model in which the elastic properties of the polymer depend explicitly on the coverage: the ratio between the monomers of the polymer occluded by ligands and the total number of monomers forming the polymer. This model is based on the effective decomposition of the polymer on its covered and uncovered regions, where the covered regions are the set of monomers occluded by the bound ligands.

Furthermore, another important issue is the understanding of the energetics of the binding process, which may allow the estimation of the coverage of the polymer. Previous studies [4,8,9,49–52] showed that the equilibrium coverage in polymers can be estimated in single-molecule manipulation experiments by comparing the elastic properties of the polymer before and after the ligand addition. These studies also showed that the coverage of the polymer depends on the concentration of the ligand, the magnitude of the stretching longitudinal force, and the ionic conditions of the solutions. In the present study, we obtain an analytic expression for the equilibrium coverage to analyze the resulting extension versus force data in single-molecule manipulation experiments. Finally, we also model the kinetics of ligand binding to the polymer.

The organization of the paper is the following: In Sec. II we introduce the aforementioned mechanic model, based on the effective decomposition of the polymer in covered and uncovered regions, and we obtain its corresponding force-extension relation. In Sec. III we employ this mechanical model to study the energetics of the system, and compute the equilibrium number of bound ligands as function of the applied force. In Sec. IV we comment the different kinds of possible transient extension effects that can appear during the binding kinetics, remarking transitory shortening or lengthening of the polymer that might arise provided different binding modes of the ligands to the polymer exist. Finally, in Sec. V we briefly summarize the main results.

## II. Mechanics: The force-extension relation

In the present study, we introduce a method to model how binding of small ligands to a biopolymer modifies its elastic properties varying the polymer chain extension at a given force depending on the coverage (number of ligands bound to the polymer).

In our approach, we assume that the extension at a given force is given by two contributions: *x*_*n*_, which represents the extension of the uncovered (or naked) regions of the polymer, and *x*_*c*_, which represents the extension of the regions of the polymer covered by ligands. Then, the total extension of the polymer, when a stretching force *F* is applied at its ends, is

$$x(F)=x_{n}(F)+x_{c}(F).$$ (1)

**Fig 1:**
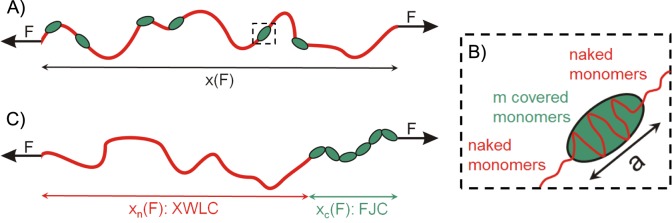
Effective model for the mechanical properties of a partially covered polymer. (Panel A) Scheme of a polymer (red), partially covered by ligands (green), under a tension *F*. (Panel B) Zoom of a ligand bound to a polymer. Each ligand covers *m* monomers, and the end-to-end distance of the DNA segment covered by one ligand is given by the parameter *a*. (Panel C) Effective mechanical decomposition of the partially covered polymer in two chains: one chain corresponds to the naked region and the other chain corresponds to the covered region. Note that distribution of ligands along the polymer is important for thermodynamic properties, this effective mechanical decomposition was done to effectively compute the extension. An extensible worm-like chain (XWLC) model is considered for the naked region, while a freely-jointed chain (FJC) model is assumed for the covered region.

Thus, the polymer can be divided into two sub-chains or regions and separately compute their extension, in order to find the total extension (see Fig 1).

Here, we assume for the naked contribution an extensible worm-like chain model (XWLC), while for the covered contribution we assume a freely-jointed chain model (FJC). This is expected to be a good model for disperse ligand binding, in which ligands orientation are random (no cooperativity binding) and the ligand remains unchanged. The key point is that if the orientations of the ligands are random, we can describe their contribution to the extension with a FJC model, which considers a polymer assembled by randomly oriented segments. If in addition the polymer orientation behind and in front of the ligand match, we can put together the uncovered polymer sections and the ensemble will have the same extension as a polymer formed with all the uncovered monomers. More generally, this approximation will hold if the changes in the polymer orientation after the ligand interaction average to zero. For example, when the ligands can diffuse over the polymer, as described for single-stranded DNA binding proteins (SSB) [14,47,53,54]. Therefore, for ligand-polymer systems following these conditions, our approximate model can be valid beyond the disperse ligand regime.

### Extension contribution of naked monomers

**Fig 2:**
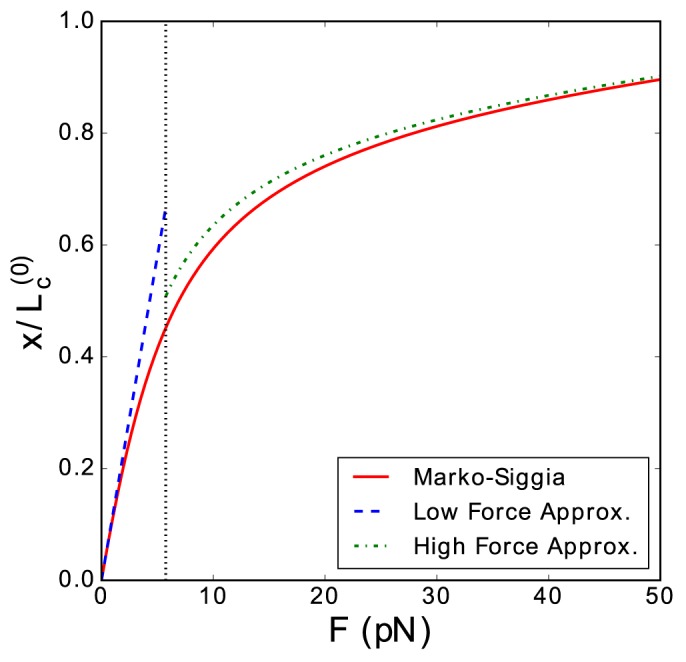
Extensions per contour length of a XWLC as function of the pulling force. Comparison of the extensions per contour length of a XWLC as function of the pulling force given by: the Marko-Siggia implicit equation (solid red line), the analytic approximations valid for the low force regime (dashed blue line), and the analytic approximation for the high force regime (dot-dashed green line). The dotted black vertical line marks the force which splits the low and high force regimes, *K*_*B*_*T*/*L*_*p*_. This figure corresponds to the the following sets of parameters: *L*_*p*_ = 0.715 *nm*, *K*_0_ = 700 *pN* (which are of the order of the values for ssDNA [24]) and *K*_*B*_*T* = 4.11 *pN nm*.

The extension of the uncovered regions of the polymer are described by the extensible worm-like chain (XWLC) model, characterized by the contour length at zero pulling force $L_{c}^{\left(0\right)}$, the persistence length *L*_*p*_, and the Young modulus *K*_0_, which represents how extensible is the naked sub-chain. The force-extension relation of the XWLC model has to be computed numerically, because it does not have an exact analytic expression. However, Wang et al. [55] obtained an implicit formula, a generalization of the Marko-Siggia formula [32] for non-extensible worm-like chains [56,57], which approximates the numerical result for all the range of forces,

$$\frac{F\;L_{p}}{K_{B}T}=\frac{1}{4}{\left(1-\frac{x_{n}(F)}{L_{c}^{\left(0\right)}}+\frac{F}{K_{0}}\right)}^{-2}-\frac{1}{4}+\frac{x_{n}(F)}{L_{c}^{\left(0\right)}}-\frac{F}{K_{0}},$$ (2)

being *K*_*B*_ the Boltzmann constant (*K*_*B*_ ≃ 8.62 ∙ 10^−5^ eV/K), and *T* the absolute temperature. One can obtain explicit analytic approximations of *x*_*n*_(*F*) for the low and the high force regimes [58],

$$\frac{x_{n}(F)}{L_{c}^{\left(0\right)}}\approx \left\{ \begin{array}{ll} \frac{2}{3}\frac{F\;L_{p}}{K_{B}T} & for\;F\ll \frac{K_{B}T}{L_{p}},\\ 1-\frac{1}{2}\sqrt{\frac{K_{B}T}{F\;L_{p}}}+\frac{F}{K_{0}} & for\;F\gg \frac{K_{B}T}{L_{p}}, \end{array}\right.$$ (3)

respectively. Fig 2 shows a comparison between the force extension relation given by the Marko-Siggia implicit formula and the force-extension relation given by the analytic approximations. These are good approximations except for the region near the force *K*_*B*_*T*/*L*_*P*_ (represented as a vertical dotted line in Fig 2), which is the boundary between the low and high force regimes.

From Eq (2) or Eq (3), it is possible to obtain the contribution to the extension of the naked regions. However, in these expressions the number of bound ligands (or, equivalently, the coverage of the chain) does not appear explicitly, but implicitly in the contour length of the naked region $L_{c}^{\left(0\right)}$,

$$L_{c}^{\left(0\right)}\equiv (N-n\;m)d_{0},$$ (4)

where *N* denotes the total number of monomers forming the polymer, *d*_0_ the contour length of the naked sub-chain per uncovered monomer, *n* the number of ligands bound, and *m* the number of monomers occluded by each ligand. This expression assumes that every ligand occludes always the same number of monomers. However, for several biological ligands this may not be the case. Multiple binding sites in a sole ligand may give raise to different binding modes, i.e., different number of monomers bound per ligand [59]. Provided there are N possible binding modes, and *n*_*i*_ ligands are bound to the polymer in mode *i*, which occludes *m*_*i*_ monomers, the contour length of the naked region would become

$$L_{c}^{\left(0\right)}=\left(N-\sum_{i=1}^{N}n_{i}\;m_{i}\right)d_{0},$$ (5)

Replacing Eq (5) in Eqs (2) or (3), we finally obtain an expression for the extension contribution of the naked regions of the polymer, which explicitly depends on the number of ligands.

### Extension contribution of covered monomers

For the covered regions of the polymer, we propose a freely-jointed chain (FJC) model, which depends on two parameters: the number of rods of the covered sub-chain *n* (number of complexes ligands-occluded monomers) and the Kuhn length *a* (effective size of the complex ligand-occluded monomers). According to the FJC model [60], the analytical expression for the Force-extension relation is

$$x_{c}(F)=n\;a\left[coth(\frac{F\;a}{K_{B}T})-\frac{K_{B}T}{F\;a}\right],$$ (6)

This expression considers a unique binding mode of the ligand to the polymer. For the case of several binding modes, we obtain the generalized expression

$$x_{c}(F)=\sum_{i=1}^{N}n_{i}\;a_{i}[\coth\left(\frac{F\;a_{i}}{K_{B}T}\right)-\frac{K_{B}T}{F\;a_{i}}],$$ (7)

where it is assumed that the covered region can effectively be divided into N different sub-chains, one for each binding mode, and the extension contribution of the covered region is just the sum of the extension contributions of all these sub-chains.

### Extension of a partially covered polymer

In our approximation, the extension of a partially covered polymer as function of stretching force is the sum of the extension contributions of the naked and covered regions. For the naked region, the implicit formula in Eq (2) or the explicit approximations in Eq (3) give the extension per contour length, with the contour length given by Eq (5). For the covered region, the extension contribution corresponds to the expression shown in Eq (7). Hence, when a tension *F* is applied, the total extension of the polymer is

$$x(F)=\frac{x_{n}(F)}{L_{c}^{\left(0\right)}}\left(N-\sum_{i=1}^{N}n_{i}\;m_{i}\right)d_{0}+\sum_{i=1}^{N}n_{i}\;a_{i}[coth(\frac{F\;a_{i}}{K_{B}T})-\frac{K_{B}T}{F\;a_{i}}].$$ (8)

In terms of the coverages, *c*_*i*_ = *n*_*i*_ *m*_*i*_/*N*, *i*.*e*., the fraction of monomers covered by each binding mode, the total extension can be rewritten as

$$x(F)=N\left\{\frac{x_{n}(F)}{L_{c}^{\left(0\right)}}(1-\sum_{i=1}^{N}c_{i})d_{0}+\sum_{i=1}^{N}c_{i}\frac{a_{i}}{m_{i}}[coth(\frac{F\;a_{i}}{K_{B}T})-\frac{K_{B}T}{F\;a_{i}}]\right\}.$$ (9)

These expressions are valid for a given polymer at a fixed temperature.

**Fig 3:**
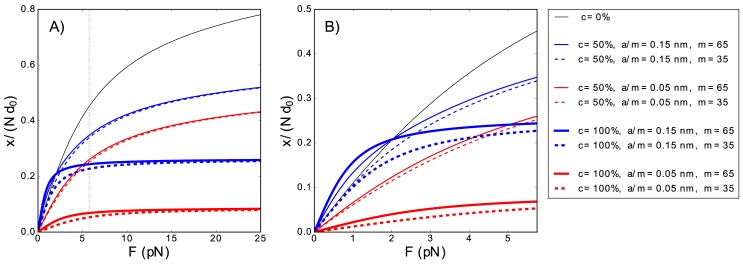
Extension of partially covered polymers as function of the pulling force. (Panel A) Extension of a polymer in the presence of a ligand as a function of the applied force for different coverages *c*, occluded monomers per ligand *m*, and *a*/*m* ratios, for a ligand with a single binding mode. The dotted vertical line marks the force value *K*_*B*_ *T*/*L*_*p*_, which separates the low and the high-force regime. In the high-force limit ($F\gg \frac{K_{B}T}{L_{p}}$), the extension *x*(*F*) depends only on both *a*/*m* and *c*. Changes in the Kuhn length per occluded monomer *a*/*m* shift the curves, but do not alter the slopes. However, changes in coverage *c* do alter these slopes. (Panel B): In the intermediate-force regime ($F∼\frac{K_{B}T}{L_{p}}$), the three parameters (*c*, *a*/*m*, and *m*) have subtle effects on the extension of the polymer. In the low-force limit ($F\ll \frac{K_{B}T}{L_{p}}$) the extension *x*(*F*) is proportional to the force *F* and the proportionality constant is given in Eq (11). For both panels, *d*_0_ = 0.57 nm. *K*_0_ = 700 pN, *L*_*p*_ = 0.715 nm (which are of the order of the values for ssDNA [24]), and *K*_*B*_*T* = 4.11 pN nm, the value at 25°*C*.

In the particular case where the ligand only has a single binding mode, N=1, the total polymer extension depends on the coverage *c*, the Kuhn length per occluded monomer *a*/*m*, and on the number of occluded monomers per ligand *m*, see Fig 3. However, in the high-force regime $F\gg \frac{K_{B}T}{L_{p}}$, and $F\gg \frac{K_{B}T}{a}$, the extension depends only on the coverage *c* and the ratio *a*/*m*

$$\underset{F\to \infty }{lim}\frac{x(F)}{N\;d_{0}}=1+\left(\frac{a/m}{d_{0}}-1\right)c+(1-c)\frac{F}{K_{0}},$$ (10)

as *K*_0_, *L*_*p*_, and *d*_*o*_ are fixed for a given polymer and temperature. In order to obtain this expression, we used Eq (3) for the naked polymer contribution, $\lim_{F\to \infty }\frac{x_{n}(F)}{L_{c}^{\left(0\right)}}=1+\frac{F}{K_{0}}$, and for the covered polymer contribution *x*_*c*_(*F*) we used $\lim_{u\to \infty }\coth\left(u\right)-\frac{1}{u}=1$.

Therefore, in the high-force regime, the force independent contribution to the extension is higher than for the naked polymer, only if *a*/*m* > *d*_0_, *i*.*e*., if the Kuhn length per occluded monomer, *a*/*m*, is higher than *d*_0_, the contour length of the naked sub-chain per uncovered monomer. Otherwise, *i*.*e*., when *a*/*m* ≤ *d*_0_, the force independent contribution to the extension is smaller. Thus, in the high-force regime, an increase in *a*/*m* just shifts the extension curves without changing the slopes, see Fig 3A. Changes in coverage *c* tune the relative relevance of the naked polymer elasticity (force-dependent term) and the Kuhn length per occluded monomer (force-independent term). Thus, coverage *c* affects both the final end-to-end extensions and their force slopes.

In contrast, in the intermediate and low-force regime, the three parameters (*c*, *a*/*m*, and *m*) affect the extension of the polymer, and their effects are not easily distinguished, see Fig 3B. The initial slope of this curves can be obtained computing the low-force regime, $F\ll \frac{K_{B}T}{L_{p}}$ and $F\ll \frac{K_{B}T}{a}$, of Eq (9),

$$\underset{F\to 0}{lim}\frac{x(F)}{N\;d_{0}}=\frac{2F\;L_{p}}{3K_{B}T}\left[1+c\;(\frac{a/m}{d_{0}}\frac{a}{2L_{p}}-1)\right],$$ (11)

where we have used Eq (3) for the naked polymer contribution, and for the covered polymer contribution *x*_*c*_(*F*) we used $\lim_{u\to 0}\coth\left(u\right)-\frac{1}{u}=\frac{u}{3}$. This expression shows that the initial slope will be greater than that for the naked chain when (*a*/*m*) ∙ *a* > *d*_0_ ∙ *L*_*p*_, *i*.*e*., when the product of the Kuhn length per occluded monomer, *a*/*m*, times the Kuhn length *a* (effective size of the complex ligand-occluded monomers) is greater than the product of *d*_0_, the contour length of the naked polymer per uncovered monomer, and *L*_*p*_ the persistence length of the naked polymer. Note that greater coverage values would enhance this effect.

Fitting the experimental force extension curves to these equations allows determining the coverage and the binding mode when the effective size of the ligand is known, as we have recently shown for human mitochondrial SSB protein [61].

Note that alternative models may be required to explain the structural organization of ligand-polymer systems in which binding of disperse oriented ligands cannot be assumed (for example, for ligands presenting positive cooperative binding).

## III. Thermodynamics: Number of ligands at equilibrium

Ligand binding to a polymer proceeds until equilibrium coverage is reached, which may depend on the force applied on the polymer. We assume that the chemical potential of *n*_*i*_ disperse ligands bound in mode *i* to the polymer at zero tension is given by the expression,

$$\mu_{i}(n_{i})=\mu_{i}^{*}+K_{B}T\;\ln\;n_{i},$$ (12)

where $\mu_{i}^{*}$ is the chemical potential of a unique ligand bound in mode *i* to the polymer at zero tension, or alternatively the ligand binding energy $\epsilon_{i}^{b}$, $\mu_{i}^{*}=-\epsilon_{i}^{b}$. The chemical potential corresponding to all the ligands bound to the polymer is assumed to be just the sum of the chemical potential for each binding mode, *μ* = ∑*μ*_*i*_(*n*_*i*_). The Gibbs free energy of the partially covered polymer is then

$$\Delta G=-\int_{0}^{F}x(\tilde{F})\;d\tilde{F}+\sum_{i=1}^{N}\int_{0}^{n_{i}}\mu_{i}({\tilde{n}}_{i})\;d{\tilde{n}}_{i},$$ (13)

This Gibbs free energy has two parts: an elastic contribution and a binding contribution. The elastic contribution has the form $-\int_{0}^{F}x(\tilde{F})\;d\tilde{F}$ for constant force; while for constant length, it has the form $\int_{0}^{x}F(\tilde{x})\;d\tilde{x}$ (with the two forms related by a Legendre transform). The binding part is the integral of the ligand chemical potentials. Using Eq (8) and Eq (10), the Gibbs free energy at tension *F* reduces to

$$\begin{array}{l} \Delta G=\left(N-\sum_{i=1}^{N}n_{i}\;m_{i}\right)d_{0}\;\Delta g_{n}^{el}(F)+\sum_{i=1}^{N}n_{i}\;a_{i}\;\Delta g_{c,i}^{el}(F)\\ \;+\sum_{i=1}^{N}n_{i}[-\epsilon_{i}^{b}{+K}_{B}T(\ln\;n_{i}-1)], \end{array}$$ (14)

where $\Delta g_{n}^{el}(F)$ and $\Delta g_{c,i}^{el}(F)$ are, respectively, the elastic contributions of the naked and covered monomers to the Gibbs free energy per unit of contour length.

$$\begin{array}{l} \Delta g_{n}^{el}(F)\equiv -\int_{0}^{F}\frac{x_{n}(\tilde{F})}{L_{c}^{\left(0\right)}}d\tilde{F},\\ \Delta g_{c,i}^{el}(F)\equiv -\int_{0}^{F}\frac{x_{c,i}(\tilde{F})}{n_{i}\;a_{i}}d\tilde{F}=-\int_{0}^{F}[\coth\left(\frac{\tilde{F}\;a_{i}}{K_{B}T}\right)-\frac{K_{B}T}{\tilde{F}\;a_{i}}]d\tilde{F}\\ \;=\;\frac{K_{B}T}{a_{i}}ln\;\left(\frac{F\;a_{i}/K_{B}T}{\sinh\left(F\;a_{i}/K_{B}T\right)}\right). \end{array}$$ (15)

Once the Gibbs free energy of the polymer is known, one can minimize it

$${{\Delta \mu }_{i}(F,n_{i})=\frac{\partial \Delta G}{\partial n_{i}}|}_{n_{i}^{\left(eq\right)}}=0,$$ (16)

giving

$$-\epsilon_{i}^{b}+K_{B}T\;\ln\left(n_{i}\right)=m_{i}d_{0}\;\Delta g_{n}^{el}(F)-a_{i}\;\Delta g_{c,i}^{el}(F),$$ (17)

and obtain the equilibrium number of ligands in mode *i* bound to the polymer at tension *F*

$$\begin{array}{l} n_{i}^{\left(eq\right)}(F)=exp\left(\frac{\epsilon_{i}^{b}+m_{i}d_{0}\Delta g_{n}^{el}(F)-a_{i}\;\Delta g_{c,i}^{el}(F)}{K_{B}T}\right)\\ \;=exp\left(\frac{\epsilon_{i}^{b}+m_{i}d_{0}\Delta g_{n}^{el}(F)}{K_{B}T}\right)\frac{\sinh\left(F\;a_{i}/K_{B}\;T\right)}{F\;a_{i}/K_{B}\;T}. \end{array}$$ (18)

**Fig 4:**
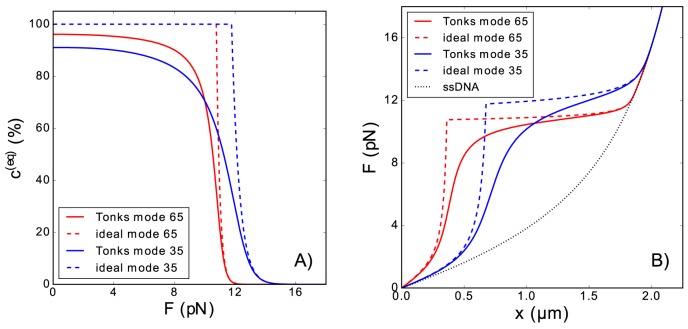
Equilibrium coverage and extension of the polymer in the presence of ligands. (Panel A): Equilibrium coverage $c_{i}^{\left(eq\right)}=n_{i}^{\left(eq\right)}\;m_{i}/N$ of a polymer as function of the tension *F*, assuming that the ligands bind to the polymer in a unique mode. The elastic contribution to the free energy of the naked monomers was estimated using the Marko-Siggia formula Eq (2). The equilibrium coverage was computed using both the ideal gas [Eq (18)] and the Tonks gas [Eq (20)] models. We also consider that the maximum possible coverage is 100%. (Panel B): Equilibrium extension of the covered polymer as function of the pulling force, resulting from employing the coverage of Panel A in the force-extension relation Eq (9). In this figure the values of the parameters of the polymer are of the order of those of ssDNA [24]: *N* = 5080, *d*_0_ = 0.57 *nm*, *L*_*p*_ = 0.715 *nm*, *K*_0_ = 700 *pN* and *K*_*B*_*T* = 4.11 *pN nm*, while, for the ligands, we consider two different binding modes, with parameters of the order of those of E. Coli SSB [47,59] and HmtSSB proteins [61]: *m* = 65 or 35, *a* = 5 *nm* and *ϵ*^*b*^ = *m* × 0.5 × *K*_*B*_*T*.

Thus, large ligand binding energy $\epsilon_{i}^{b}$ favours binding, but at high tension the elastic energy cost of converting naked monomers in covered monomers favours unbinding. Since the elastic energy grows with tension, tension favours the transition to a naked polymer. On the one hand, in the high-force limit, *F* ≫ *K*_*B*_ *T*/*L*_*p*_, there is a quadratic relationship between the elastic contribution of the naked monomers and the pulling force, $\Delta g_{n}^{el}(F)∼-F^{2}/K_{0}$ [using Eqs (3) and (15)]. On the other hand, the covered monomer contribution $\Delta g_{c,i}^{el}(F)$ scales linearly with pulling force in the high-force limit. Therefore, assuming that naked contribution dominates, we can estimate the transition force as $∼\sqrt{\frac{\epsilon_{i}^{b}\;K_{0}}{m_{i}d_{0}}}$. If the values plotted in Fig 4 are used, this approximate expression predicts that the transition occurs at *F* ∼ 50 *pN*, while the complete Eq (18) predicts the transition occurs at *F* ∼ 10–15 *pN*, as show in Fig 4.

We have assumed that the size of the region covered by the ligand is smaller than the contour length of the polymer, and we used for the chemical potential of the bound ligands the unidimensional ideal gas form, Eq (12). This expression allowed obtaining a simple analytical estimation of the transition force. However, at high coverage, the change in the entropic contribution of the uncovered regions will become important, and the Tonks gas expression [62] will be a more accurate description. An analogous computation gives the following implicit relation for the coverage as a function of the force, for the case of a single binding mode

$$-\epsilon^{b}+K_{B}T\;\left[\ln\left(\frac{N}{m}\right)+\ln\left(\frac{c}{1-c}\right)+\frac{c}{1-c}\right]=m\;d_{0}\;\Delta g_{n}^{el}(F)-a\;\Delta g_{c}^{el}(F).$$ (19)

This equation can be solved for the coverage *c* in terms of the Lambert function *W*_0_ [63–65]

$$c(F)=\frac{1}{1+\frac{1}{W_{0}(\frac{m}{N}e^{\frac{E(F)}{K_{B}T}})}},$$ (20)

with $E(F)=\epsilon^{b}+m\;d_{0}\;\Delta g_{n}^{el}(F)-a\;\Delta g_{c}^{el}(F)$. This result is represented in Fig 4A and compared with the ideal gas result. Also in Fig 4B we combine this result with the mechanical model in Section II and represent the corresponding force extension curves for a particular case with similar parameters to the single strand binding protein (SSB) bound to single strand DNA (ssDNA), for the particular cases of E. Coli SSB [47,59] and human mitochondrial SSB [61]. These plots have the same form as those experimentally obtained for these two SSB in recent single-molecule experiments [47,61]. It is important also to notice that Tonks model predicts a maximum coverage lower than the complete coverage, see Fig 4A.

Additionally, in some cases cooperative ligand binding effects could also be present [66].

## IV. Kinetics: Binding of ligands to polymer

The interactions between ligands and biopolymers are relevant to fundamental biological processes. For example, ligand binding to a biopolymer can change its mechanical (including polymer extension, or end-to-end distance) and/or chemical properties interfering in this way with its biological functions. Thus, understanding the influence of the binding kinetics on the elastic properties of biopolymers is essential. In this section, we analyze the time evolution of the coverage of a polymer by ligands in two common scenarios: one binding mode and two binding modes. In addition, we discuss the relation between the coverage of the polymer and its extension.

### One binding mode

First, we study the case of a unique binding mode of the ligand to the polymer, where the ligand attaches to *m* monomers. In this case, the number of ligands attached to the polymer will vary following the differential equation

$$\frac{dn}{dt}=k_{b}\frac{N-n\;m}{m}-k_{r}\;n,$$ (21)

where *k*_*b*_ stands for the binding rate of the ligands to the polymer, which is multiplied by the *number of holes* (naked regions of length m) present in the polymer, and *k*_*r*_ stands for the releasing rate of the ligands from the polymer, which is multiplied by the number of bound ligands. The concept of *number of holes* has been defined as the number of naked monomers of the polymer divided by the number of monomers that a single bound ligand occludes, so it represents the number of extra ligands needed to completely cover the polymer. Assuming that at time *t* = 0 there is not any ligand attached to the chain, and then we add a certain ligand concentration into the buffer, the number of bound ligands at each time is

$$n(t)=\frac{N}{m}\frac{1}{1+\frac{k_{r}}{k_{b}}}(1-e^{-(k_{b}+k_{r})t}).$$ (22)

**Fig 5:**
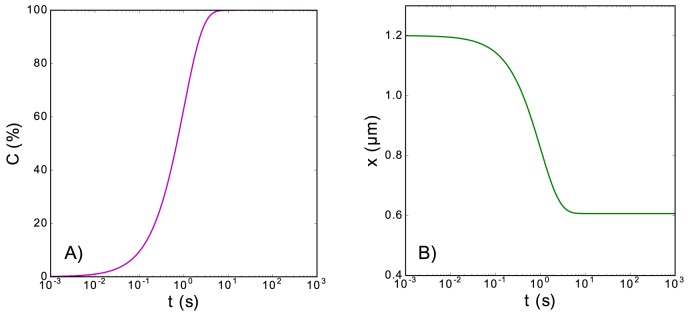
Ligand binding evolution for a unique binding mode. (Panel A): Time evolution of the coverage of the polymer when there is a unique binding mode. (Panel B): Temporal evolution of the extension corresponding to the evolution of the coverage shown in Panel (A). Both panels correspond to the following set of parameters: *k*_*b*_ = 1 *s*^−1^, *k*_*r*_ = 10^−5^ *s*^−1^, *N* = 5080, *m* = 35, *d*_0_ = 0.57 *nm*, *L*_*p*_ = 0.715 *nm*, *K*_0_ = 700 *pN*, *a* = 5 *nm*, *K*_*B*_*T* = 4.11 *pN nm*, and *F* = 5 *pN*. For the extension contribution of the naked monomers, we have employed the Marko-Siggia implicit formula [Eq (2)].

As we can see from this expression, the equilibrium number of ligands bound to the polymer is *N*/[*m* (1 + *k*_*r*_/*k*_*b*_)], or equivalently, the equilibrium coverage of the polymer is 1/(1 + *k*_*r*_/*k*_*b*_), which is reached in the limit *t* → ∞. Fig 5 shows an example of the time evolution of the coverage (Panel A) and the extension (Panel B) of the polymer.

### Two binding modes

The binding kinetics is quite simple when the ligand has a unique binding mode. However, for several binding modes, the kinetics is more complex. Furthermore, since different binding modes compact the polymer in different ways, this might lead to the appearance of transitory shortening or lengthening of the chain. These transient effects can arise for different causes, as we show below.

#### Transient effects due to competition between fast and slow binding modes

For the sake of simplicity, we will assume that there are just two different binding modes, labelled modes 1 and 2, with different binding rates, *k*_*b*1_ and *k*_*b*2_, and release rates, *k*_*r*1_ and *k*_*r*2_. The binding kinetics equations are

$$\begin{array}{l} \frac{dn_{1}}{dt}=k_{b1}\frac{N-n_{1}\;m_{1}-n_{2}\;m_{2}}{m_{1}}-k_{r1}n_{1},\\ \frac{dn_{2}}{dt}=k_{b2}\frac{N-n_{1}\;m_{1}-n_{2}\;m_{2}}{m_{2}}-k_{r2}n_{2}. \end{array}$$ (23)

**Fig 6:**
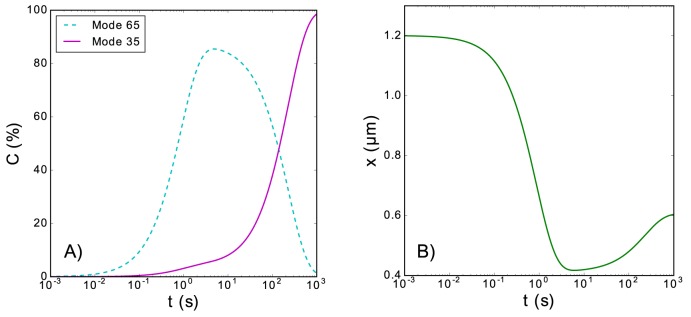
Transient length shortening due to binding mode competition. (Panel A) Temporal evolution of the coverage of the polymer during the binding process when there are two binding modes with different temporal scales: a fast mode 1 and a slow mode 2. (Panel B): Temporal evolution of the extension corresponding to the evolution of the coverage shown in panel (a). This figure corresponds to the following set of parameters: *k*_*b*1_ = 1 *s*^−1^, *k*_*b*2_ = 5 ∙ 10^−2^ *s*^−1^, *k*_*r*1_ = 0.1 *s*^−1^, *k*_*r*2_ = 10^−5^ *s*^−1^, *N* = 5080, *m*_1_ = 65, *m*_2_ = 35, *d*_0_ = 0.57 *nm*, *L*_*p*_ = 0.715 *nm*, *K*_0_ = 700 *pN*, *a*_1_ = *a*_2_ = 5 *nm*, *K*_*B*_*T* = 4.11 *pN nm*, and *F* = 5 *pN*. For the extension contribution of the naked monomers, we have employed the Marko-Siggia implicit formula [Eq (2)].

If we assume that the binding rate of mode 1 is much faster, *k*_*b*1_ ≫ *k*_*b*2_, the ligand will first bind to the polymer mainly in mode 1. After if the binding is stronger in mode 2, $\frac{k_{b2}}{k_{r2}}\ll \frac{k_{b1}}{k_{r1}}$, the ligands in mode 1 will release the polymer and be replaced by ligands in mode 2. This phenomenon of transient coverage by mode 1 is illustrated in Fig 6, where we also show that it can be observed as a transient change in extension of the polymer. Initially, ligands bind to the polymer in mode 1, due to its larger binding rate, but at the end, most of the ligands on the polymer are bound in mode 2, due to its stronger binding energy. This transient coverage in a different mode may cause a transitory shortening of the polymer. For the example in Fig 6, the extension of the polymer first shortens when the ligands bind in mode 1, and then lengthens when the ligands bound in mode 1 are replaced by ligands bound in mode 2.

#### Transient effects due to conversion between binding modes

These transitory changes in mode coverage and length can have another source, the direct conversion of modes without an intermediate release. Assume that the ligands bound to the polymer can change its binding mode. Also assume that this change of mode can just happen in sets of *s* ligands, and that in mode 1 the ligands occlude more monomers than in mode 2 (*m*_1_ > *m*_2_). Then, we can model the binding kinetics with the system of differential equations

$$\begin{array}{l} \frac{dn_{1}}{dt}=k_{b1}\frac{N-n_{1}\;m_{1}-n_{2}\;m_{2}}{m_{1}}-k_{r1}\;n_{1}-s\;k_{1\to 2}\;n_{1}^{s}\\ \;+k_{2\to 1}\;n_{2}^{s}\frac{N-n_{1}\;m_{1}-n_{2}\;m_{2}}{m_{1}-m_{2}}\\ \frac{dn_{2}}{dt}=k_{b2}\frac{N-n_{1}\;m_{1}-n_{2}\;m_{2}}{m_{2}}-k_{r2}\;n_{2}+s\;k_{1\to 2}\;n_{1}^{s}\\ \;-k_{2\to 1}\;n_{2}^{s}\frac{N-n_{1}\;m_{1}-n_{2}\;m_{2}}{m_{1}-m_{2}} \end{array}$$ (24)

**Fig 7:**
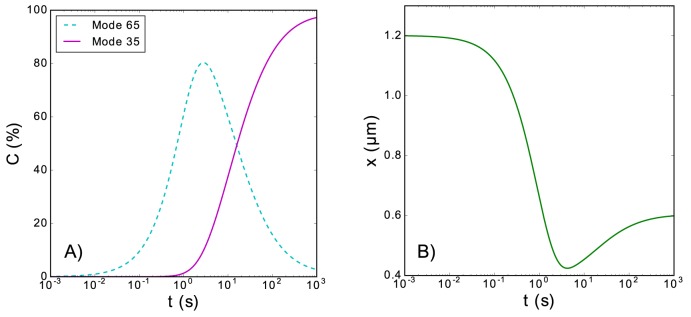
Transient length shortening due to conversion between binding modes. (Panel A): Temporal evolution of the coverage of the polymer when there are two binding modes of the ligands bound to the polymer, and the bound ligands can change its mode in sets of *s* ligands. (Panel B): Temporal evolution of the extension corresponding to the evolution of the coverage shown in Panel A. This figure corresponds to the following set of parameters: *s* = 2, *k*_*b*1_ = 1 *s*^−1^, *k*_*b*2_ = 0 *s*^−1^, *k*_*r*1_ = *k*_*r*2_ = 10^−5^ *s*^−1^, *k*_1→2_ = 10^−3^ *s*^−1^, *k*_2→1_ = 5 ∙ 10^−5^ *s*^−1^, *N* = 5080, *m*_1_ = 65, *m*_2_ = 35, *d*_0_ = 0.57 *nm*, *L*_*p*_ = 0.715 *nm*, *K*_0_ = 700 *pN*, *a*_1_ = *a*_2_ = 5 *nm*, *K*_*B*_*T* = 4.11 *pN nm*, and *F* = 5 *pN*. For the extension contribution of the naked monomers, we have employed the Marko-Siggia implicit formula [Eq (2)].

This conversion between modes can also lead to similar transitory effects as the observed in the case of existence of slow and fast modes: first, ligands bind to the polymer in mode 1, and later these ligands change their binding mode in sets of *s* ligands. In Fig 7 we plot an example of temporal evolution of the coverage (Panel A) and the extension (Panel B), when there are two possible binding modes and the bound ligands can change their mode. The observed transitory effects are similar to the ones shown in Fig 6 for the release-mediated conversion.

This simplified linear picture for one and two binding modes is corrected by non-linearities due to overlap binding, even in the absence of cooperativity (non-interacting ligands) [66]. However, the binding rates in these linear equations can be seen as effective binding rates including the leading effects of overlap binding [66]. Fitting the predictions of the previous equations to the experimental temporal variations of extension during the binding process allows computing the effective binding, unbinding, and conversion rates.

## V. Conclusions

This paper contributes to understand the binding of ligands to long polymers, a common scenario in molecular biology. We have developed an approach to explain the mechanical, thermodynamics, and the chemical kinetics behaviors of the ligands-polymer system.

In our approach, the partially covered polymer is effectively divided into its naked and covered parts, being the extension of the polymer the sum of the extension contributions of these regions. Under this assumption, we derived the expression for the force-extension relation that explicitly depends on the coverage of the polymer by the ligands. Then, in the study of the thermodynamics of the process, we used this expression to calculate the Gibbs free energy of the ligands-polymer system. Furthermore, the equilibrium coverage of the polymer, i.e. the coverage that minimizes the free energy, is estimated as a function of the tension. Finally, we studied the kinetics of the binding process, in order to know how the coverage of an initially naked polymer evolves until reaching the equilibrium value. We show that when the ligand presents different binding modes the binding dynamics can lead to transient shortening or lengthening of the polymer due to changes or transitions between the binding modes.

The proposed model and its implications constitute an important theoretical tool for the study of ligand-polymer systems in single molecule manipulation experiments. This model provides a method to estimate ligand coverage and ligand mode from experimental force extension curves. In addition, this model allows computing binding, unbinding, and conversion rates from the temporal variations of extension during the binding process. This method would be of special relevance to study the complex interactions between proteins with ssDNA. The proposed model can be used, for example, to understand the mechanics, thermodynamics, and assembly kinetics of ssDNA-SSB complexes. The great flexibility of ssDNA and large heterogeneity of the protein-DNA interface has hindered the advance of the research in this field. The model and the associated method described here can be applied beyond the disperse ligand regime, as shown in single molecule manipulation studies with the HmtSSB [61].

The model gives a simple effective description of ligand-polymer systems. Further details as finite size effects, motility of ligands and cooperativity effects may be relevant to explain the properties of other systems.
